# Correlated Spontaneous Activity Persists in Adult Retina and Is Suppressed by Inhibitory Inputs

**DOI:** 10.1371/journal.pone.0077658

**Published:** 2013-10-29

**Authors:** Abduqodir H. Toychiev, Christopher W. Yee, Botir T. Sagdullaev

**Affiliations:** 1 Department of Neurology, Weill Medical College of Cornell University, New York, New York, United States of America; 2 Department of Ophthalmology, Weill Medical College of Cornell University, New York, New York, United States of America; Morehouse School of Medicine, United States of America

## Abstract

Spontaneous rhythmic activity is a hallmark feature of the developing retina, where propagating retinal waves instruct axonal targeting and synapse formation. Retinal waves cease around the time of eye-opening; however, the fate of the underlying synaptic circuitry is unknown. Whether retinal waves are unique to the developing retina or if they can be induced in adulthood is not known. Combining patch-clamp techniques with calcium imaging, we demonstrate that propagative events persist in adult mouse retina when it is deprived of inhibitory input. This activity originates in bipolar cells, resembling glutamatergic stage III retinal waves. We find that, as it develops, the network interactions progressively curtail this activity. Together, this provides evidence that the correlated propagative neuronal activity can be induced in adult retina following the blockade of inhibitory interactions.

## Introduction

Correlated spontaneous activity is present during the development of various parts of the nervous system, including the spinal cord, the hippocampus, and the retina [1]. Retinal ganglion cells (GCs), the neurons responsible for transmitting visual signals from the eye to the higher brain, exhibit rhythmic bursts of action potentials that propagate across the developing retina, known as retinal waves. Retinal waves are crucial to the formation of the visual system, playing a central role in eye-specific segregation within the lateral geniculate nucleus (LGN), as well as retinotopic map formation in the superior colliculus, LGN, and visual cortex [2].

The cessation of waves is a landmark of visual development, occurring around the time of eye-opening [2]; however, it remains unclear whether the mechanisms that generate waves are irreversibly deactivated late in development, or if they persist under active suppression. A number of indirect observations suggest that the latter may be true. Though waves are not present in adult retina, a variety of cells exhibit rhythmic activity [3], [4], [5], which is suppressed by the inhibitory network [6]. Similarly, it has been suggested that the cessation of retinal waves is related to the maturation of the inhibitory network [7], [8]. GABA and glycine receptors differently modulate retinal waves at progressive stages of retinal development [9], [10], [11], [12], due to a gradual transition from being excitatory to being inhibitory [13], [14], [15]. While untested, the presence of spontaneous oscillatory activity after blocking inhibitory transmission suggests that a mature inhibitory network suppresses wave-generating mechanisms during adulthood.

Here, we provide evidence that wave-generating mechanisms exist in adulthood and can be unveiled by blocking inhibitory transmission. First, we use electrophysiological recordings in wholemount retinal tissue to reveal spontaneous regenerative activity in retinal bipolar cells. Second, we use calcium imaging to show that the block of the inhibitory network unveils coordinated cellular events that propagate across the retina in waves. We then dissect the contributions of gap junction signaling, glutamate spillover, and voltage-gated sodium channels to wave propagation. Finally, we identify the unique roles of individual components of the inhibitory network that modulate and ultimately suppress this activity in adult retina. These experiments demonstrate that the generation of spontaneous propagating activity is not a transient mechanism that is restricted to retinal development. Instead, wave-generating machinery exists in adulthood, where it is silenced by a mature inhibitory network.

## Methods

### Ethics Statement

In all experimental procedures presented in this study, the animals were treated according to the regulations in the ARVO Statement for the Use of Animals in Ophthalmic and Vision Research and in compliance with a protocol approved by the Institutional Animal Care and use Committee (IACUC) of Weill Cornell Medical College (Protocol #0809-785A). Wild type mice (C57BL/6J) of either sex were purchased from Jackson Labs (Bar Harbor, ME).

### Preparation of Retina Wholemounts

Methods for wholemount electrophysiology and calcium imaging have been previously described [16]. After the animal was euthanized, its eyes were enucleated and placed in oxygenated HEPES-buffered extracellular solution, containing (in mM) 137 NaCl, 2.5 KCl, 2.5 CaCl_2_, 1.0 MgCl_2_, 10 Na-HEPES, 28 glucose, pH 7.4. The cornea, iris and lens were removed with small scissors. The retina was dissected into four equal quadrants. Using gentle suction, each quadrant was attached photoreceptor surface down to a modified translucent Millipore Millicell filter ring (Millipore, Bedford, MA). Individual wholemounts were transferred to a recording chamber on the stage of an upright Nikon FN1 microscope equipped with Hoffman modulation contrast optics (Modulation Optics, Inc.; Greenvale, N.Y.), as described previously [16]. The recording chamber was constantly superfused (1 ml/min) with bicarbonate-buffered Ames solution (Sigma, St.Louis, MO), and was bubbled with 95% O_2_ and 5% CO_2_. Pharmacological agents were added to the control solution and bath applied using an eight-channel superfusion system (Warner Instruments, Hamden, CT). Reagents including 6-cyano-7-nitroquinoxaline-2,3-dione (CNQX), 1,2,5,6-tetrahydropyridine-4yl) methyphosphinic acid (TPMPA), strychnine hydrochloride, nifedipine, mibefradil dihydrochloride hydrate, bicuculline methbromide, Lidocaine N-ethyl bromide (QX-314) were obtained from Sigma; D(-)-2-amino-5-phosphonopentanoic acid (D-AP5), and SR95531 hydrobromide (gabazine) were obtained from Tocris (Ballwin, MO).

### Recording Procedures

Bipolar cell membrane potential was measured in whole-cell current clamp mode with K^+^-based intracellular solution [17]. The location of bipolar cells within the inner retina necessitates navigating through tissue to access the cell with the recording pipette. To accomplish this while preserving a maximal number of connections to and from the bipolar cell, we created a small incision into the tissue, targeting cells at the apex. This approach is advantageous compared to those that remove superficial retinal layers or target cells at the edge of the retinal tissue. The internal solution contained (in mM):125 K-aspartate, 10 KCl, 10 HEPES, 5 N-methyl-d-glucamine (NMG)–N-hydroxyethyl-ethylene diaminetriacetic acid, 1 MgCl_2_, 0.5 CaCl_2_, 4 ATP-Mg, 0.5 GTP-Tris; pH was adjusted to 7.2 with NMG-OH, and osmolarity was ∼280 mOsm. Synaptic currents of amacrine and GCs were measured using whole-cell recordings with patch pipettes filled with intracellular solution containing (in mM): 120 Cs-gluconate, 10 tetraethylammonium chloride (TEA-Cl), 1.0 CaCl_2_, 1.0 MgCl_2_, 11 ethylene glycol-bis(beta-aminoethyl ether)-N,N,N′,N′-tetraacetic acid (EGTA), and 10 sodium N-2-hydroxyethylpiperazine-N′-2-ethanesulfonic acid (Na-HEPES), adjusted to pH 7.2 with CsOH. The calculated E_Cl_ for this solution was −58 mV. The GC spiking activity was recorded in a cell-attached mode prior to breaking the seal. All intracellular solutions were supplemented with 0.05% sulforhodamine B. Electrodes were pulled from borosilicate glass (1B150F-4; WPI, Sarasota, FL) with a P-97 Flaming/Brown puller (Sutter Instruments, Novato, CA) and had a measured resistance of ∼4–7 MΩ. All recordings were made with MultiClamp 700B patch-clamp amplifier (Molecular Devices, Sunnyvale, CA) using Signal (CED, UK) to generate voltage command outputs and acquire data. Data were filtered at 5 kHz with a four-pole Bessel filter and were sampled at 15 kHz. Temperature of the solution and the recording chamber was maintained at near physiological level of 32°C (Warner Instruments, Hamden, CT).

### Identification of Morphological Cell Types

Each recorded cell was filled with sulforhodamine B, included in patch pipette solution. At the end of the recording session, the contrast and fluorescent images of the cell were documented with a modified Nikon D5000 DSLR attached to Nikon FN1 microscope. Then the preparation was immediately placed in glass bottom culture dish (Matek, Ashland, MA) and transferred to the stage of a Nikon C-1 confocal microscope. A z-stack of 160 images was acquired at 0.5 um steps at a resolution of 1024×1024 pixels. A nuclear stain, 2 µL of an equal mixture of 12 mM ethidium bromide and 100 µM ToPro-3 (Invitrogen, Carlsbad, CA) was added for determining the borders of the inner plexiform layer (IPL) and the depth of the dendritic stratification. BCs were classified following the criteria outlined in [18]. GCs were distinguished from displaced amacrine cells by the presence of an axon. The level at which the dendritic processes stratified in the IPL was measured as distance from its processes from proximal (0%) to the distal margin (100%) of the IPL. In general, ON cells were defined as those whose dendrites stratified <60% of the IPL depth, and OFF cells stratified >60% of the IPL depth.

### Calcium Imaging

Retinas were loaded with the calcium indicator Oregon Green 488 BAPTA-1 AM (OGB-1 AM; Invitrogen) using the multicell bolus loading technique [19]. OGB-1-AM stock 10 mM solution in 2% pluronic in DMSO was diluted 1/10 in a HEPES-buffered media containing (in mM) 150 NaCl, 2.5 KCl, 10 HEPES, pH 7.4. The pipette containing the OGB-1-AM was positioned under the inner limiting membrane, and dye was pressure injected for 1 s in three to five locations per retina at a pressure of 10–20 psi (Picospritzer III, Parker Automation). Epifluorescent calcium imaging was performed on an inverted Nikon Eclipse Ti-U microscope with 20× water-immersion objective (Nikon PlanFluor). Images were acquired at 24 frames per second using a custom modified Nikon D5000 digital camera. Video frames were imported into Matlab (Mathworks, Natick, MA) for ΔF/F analysis using custom-written scripts. ΔF/F calculations were performed on a pixel-by-pixel basis, using the initial frame as a baseline. Subsequent frames were averaged in pairs to optimize memory allocation and reduce interframe video noise artifacts from the imaging sensor, producing 12 ΔF/F frames per second. ΔF/F values were mapped to a color gradient for display purposes. The illustrations shows the full color gradient, while supporting videos utilize a minimum threshold of 50% of the maximum ΔF/F to only include higher intensity values, which are run through a noise filter and overlaid on the original video frames. Velocities were calculated using the leading edge of waves, which were defined as contiguous moving areas of calcium activity lasting for 3 ΔF/F frames (0.25 s) or more.

## Analysis

Excitatory and inhibitory postsynaptic currents (EPSCs and IPSCs, respectively) were collected, averaged and analyzed using Signal software (Cambridge Electronic Design, Ltd., UK). To quantify the strength of synaptic oscillations, the power spectra of EPSCs and IPSCs traces were obtained using a Hanning window, and had a resolution of 0.076 Hz bins. Frequency of oscillations was determined by finding the peak power within the range of 0.1 and 30 Hz, and power was measured by summing the area under the curve at the peak frequency. Following SST treatment, we did not observe oscillations below 0.1 Hz, and none exceeded 2 Hz [6]. Oscillatory activity in rd mutants has been observed between 3–20 Hz [6], [20], [21]. The frequency range was chosen to accommodate these previous studies and current observations. Heat map spectrograms were generated for consecutive recording frames using custom scripts written in Matlab (Mathworks, Natick, MA). Statistical analyses were performed using SigmaPlot (Systat Software Inc. Richmond, CA) and SPSS (version 17, SPSS Inc., Chicago, IL). All data are reported as means ± SEM. Statistical significance was determined either using a Student’s t-test or paired t-test or, for multiple comparisons, using one-way ANOVA and Bonferroni post-hoc. The Kolmogorov-Smirnov test was used for distribution comparisons.

## Results

The following experiments examine wave-like activity in the adult retina. Our work demonstrates that (i) spontaneous regenerative activity is revealed in BCs following inhibitory blockade, and is transmitted to postsynaptic cells; (ii) this activity propagates across the retina; (iii) distinct components of the inhibitory network differently shape this activity.

### Block of Tonic Inhibition Unveils Spontaneous Regenerative Activity of Bipolar Cells

**Figure 1:**
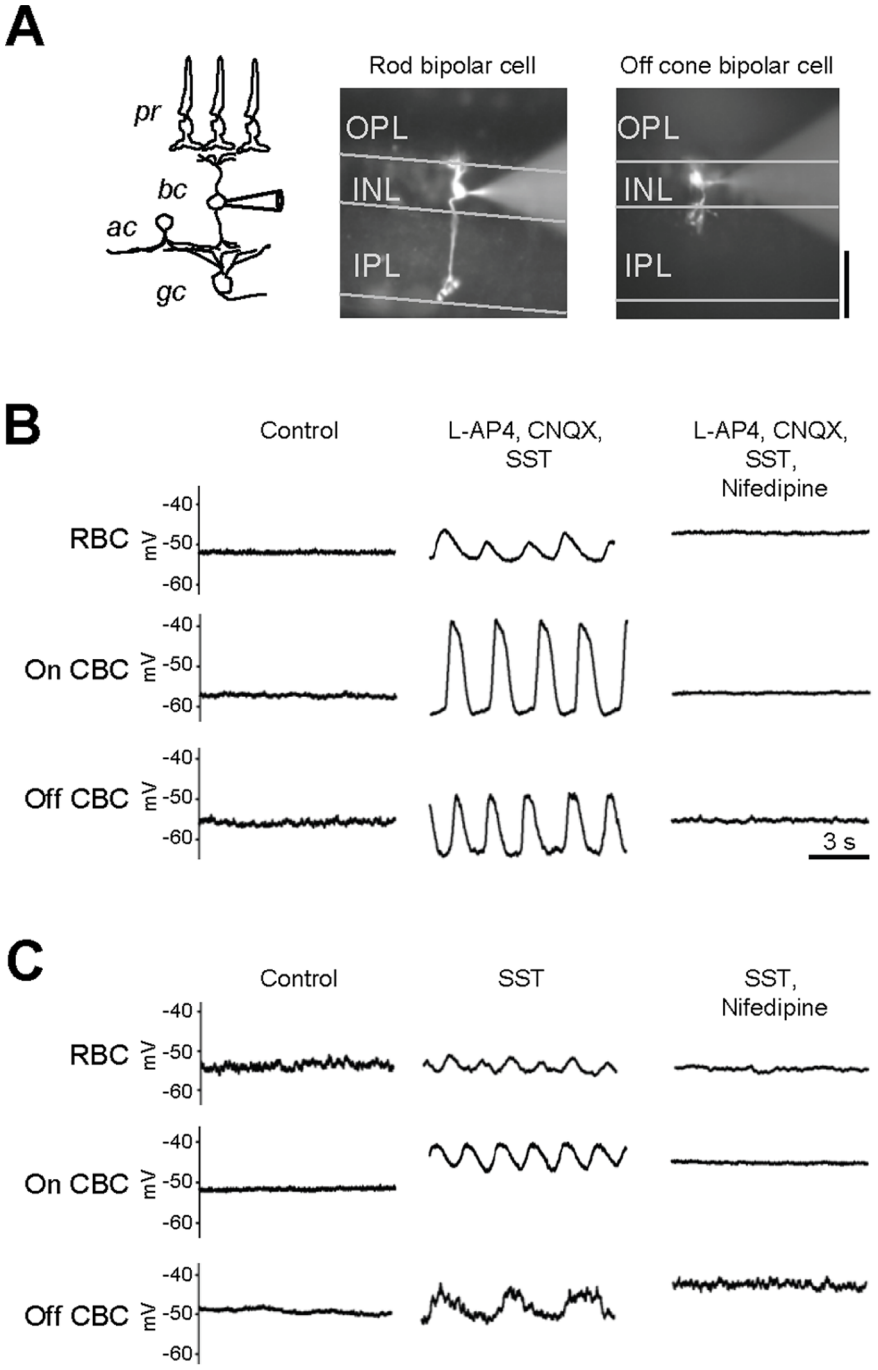
Synaptic inputs suppress regenerative activity in adult retinal bipolar cells. A: Schematic of a basic retinal circuitry with fluorescence images of representative BCs in the retina (P35–65). Horizontal lines define retinal synaptic and nuclear layers. pr - photoreceptor, bc - bipolar, ac -amacrine and gc - ganglion cells. B: The membrane potentials of identified BCs at rest and after blockade of dendritic and axonal synaptic inputs (middle) and L-type VGCaC (right). C: The membrane potentials at rest and after blockade of axonal synaptic inputs (middle) and L-type VGCaC (right).

Late in development, retinal waves rely on glutamatergic transmission from BCs [22], [23], [24]. To determine whether this spontaneous regenerative activity persists in adulthood, we targeted BCs in wholemount retina from adult (P35–P65) mice and recorded membrane potentials under conditions of synaptic blockade. Cells were backfilled with fluorescent dye, and visualized using confocal microscopy. BCs were identified as rod, on-cone, or off-cone BCs based on the shape and depth of arbor stratification in the IPL (Fig. 1A). Blockade of both dendritic and axonal synaptic inputs – using a pharmacological cocktail consisting of the mGluR6 agonist L-AP4 (20 µM), the iGluR antagonists CNQX/DNQX (10 µM), the glycine receptor (GlyR) antagonist strychnine (5 µM), and the GABA_A_ and GABA_C_ receptor antagonists SR-95531 (10 µM) and TPMPA (100 µM) – induced spontaneous regenerative activity in all recorded BCs (n = 4 out of 4, for each cell type; Fig. 1B). This is consistent with observations in dissociated BCs [3], [4], and suggests that regenerative activity is an intrinsic property of BCs, which is suppressed by synaptic inputs in situ.

Inhibitory inputs to BCs shape response properties during both development [9], [10], [14], [15] and adulthood [25]. The removal of inhibition leads to the occurrence of oscillatory activity in GCs [6], suggesting that BCs are the source of this activity. While a subset of amacrine cells can also release glutamate [26], [27], they do not contribute to glutamatergic oscillatory activity [24]. Thus, blocking inhibition to BCs alone is expected to unveil regenerative activity. Indeed, a cocktail of SR-95531 (10 µM), strychnine (5 µM) and TPMPA (100 µM) (SST) led to ∼5–10 mV membrane potential oscillations in BCs (Fig. 1C, rod n = 4, on-cone n = 3, and off-cone BC n = 3). Together, these data indicate that intrinsic regenerative activity is present in diverse classes of BCs in the intact adult retina, and is revealed after blocking inhibitory transmission.

### Regenerative BC Activity is Transmitted to Postsynaptic Cells, and Relies on L-type Voltage-gated Calcium Channels

**Figure 2:**
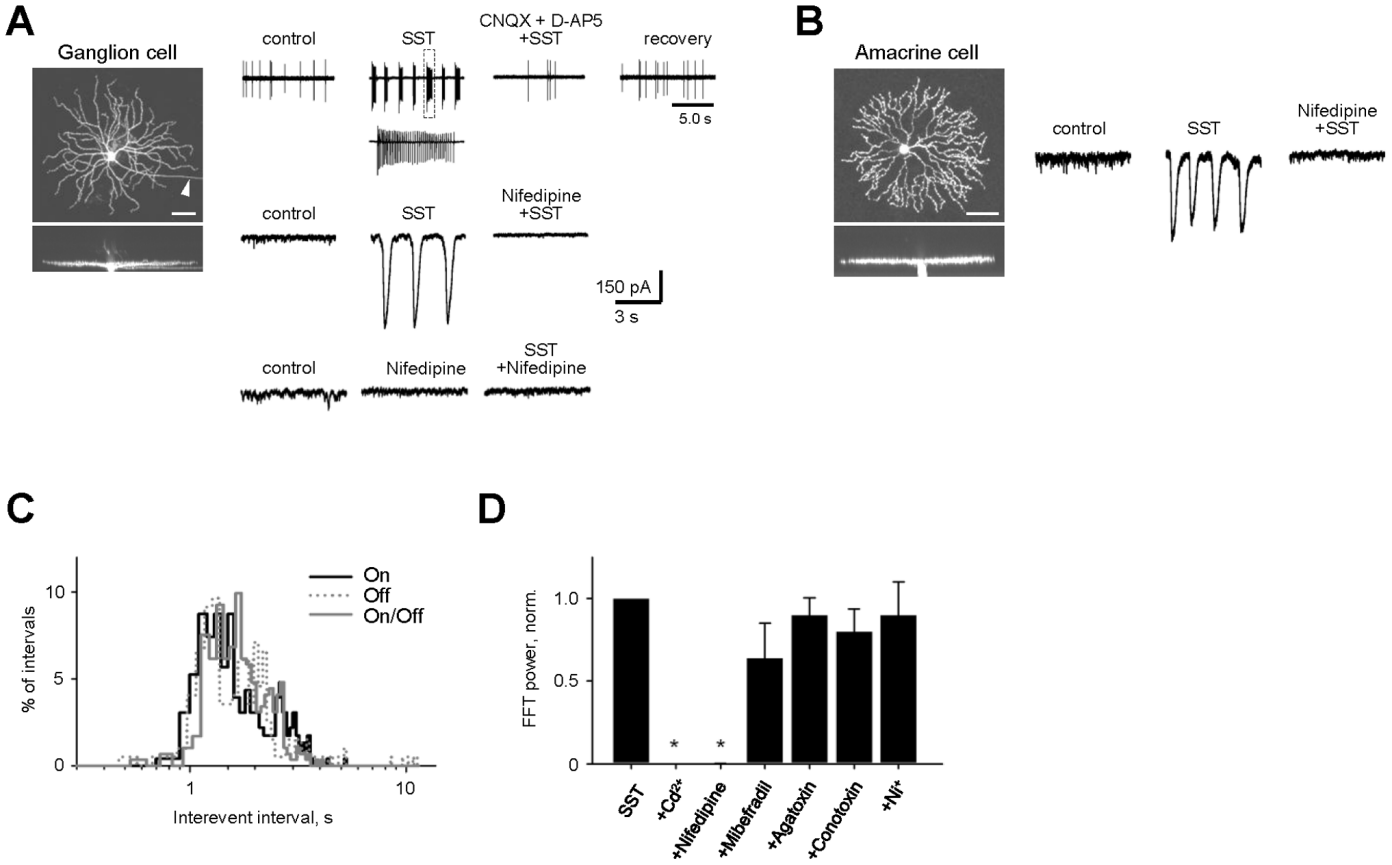
Large compound events are present in postsynaptic ganglion and amacrine cells and rely on inputs from BCs and L-type voltage gated calcium channels (VGCaCs). A and B: Spiking activity and sEPSCs from (A) a G2 ganglion cell [72] and (B) a starburst amacrine cell in P35 retina. Application of inhibitory receptor blockers strychnine, SR-95531 and TPMPA (SST) induced large-amplitude oscillatory activity, which could be eliminated by either blocking the excitatory inputs with CNQX and D-AP5 or VGCaCs with nifedipine. C: Distribution of interevent intervals shows no significant difference in frequency of oscillatory activity across different GC classes. D: Sensitivity profile of oscillatory activity to pharmacological blockers of various types of Ca^2+^-channels (see text for details). All data are reported as means ± SEM.

Next, we tested whether the regenerative activity in adult BCs is synaptically transmitted to postsynaptic targets. We found that both postsynaptic ganglion and amacrine cells exhibited rhythmic spontaneous oscillations following SST treatment (Fig. 2A and B, n = 329). This activity was abolished by the iGluR blockers CNQX (10 µM) and D-AP5 (50 µM), demonstrating that it relies on synaptic transmission (Fig. 2A). The oscillatory currents also drove periodic bursts of spiking activity in GCs in current clamp recordings (Fig. 2A, top).

To test whether this activity differs between parallel pathways, we calculated interevent intervals for ON, OFF, and bistratified ON/OFF GCs, and found that they did not differ between these major cell classes, suggesting that BC oscillations did not preferentially affect a given retinal pathway (Fig. 2C, p>0.1, Kolmogorov-Smirnov test; n = 115, 115, 73, respectively).

Both CdCl_2_ (200 µM), a wide spectrum Ca^2+^ channel blocker, and nifedipine (30 µM), a selective voltage gated L-type Ca^2+^ channel (VGCaC) blocker, abolished spontaneous excitatory events (p<0.001 for both conditions; n = 3 and 8, respectively, ANOVA with Bonferroni post-hoc multiple comparisons). This effect was not due to disruption of synaptic transmission between a BC and its postsynaptic targets, but rather through a direct effect of nifedipine on regenerative potentials in BCs (Fig. 1C), as nifedipine is not a glutamate receptor antagonist and thus should not block synaptic transmission. This is consistent with findings in goldfish Mb1 BCs [3], [28], [29]. While this activity is dependent on L-type voltage-gated calcium channels, the necessity of other types of calcium channels remains unclear. For instance, regenerative potentials in dissociated rat BCs rely on T-type Ca^2+^ channels [4], [30]. Therefore, we screened the effect of blockers of T-, P/Q-, N-, and T/R-type Ca^2+^ channels (10 µM mibefradil, n = 5, 200 nM agatoxin IVA, n = 4, 10 nM conotoxin GVIA, n = 5, and 100 µM Ni^2+^, n = 5, respectively), and found that they did not significantly affect spontaneous oscillatory activity (p = 0.44, 0.81, 1.00, and 1.00, respectively, ANOVA with Bonferroni post-hoc multiple comparisons).

### Calcium Imaging Reveals Propagating Waves in Adult Retina

**Figure 3:**
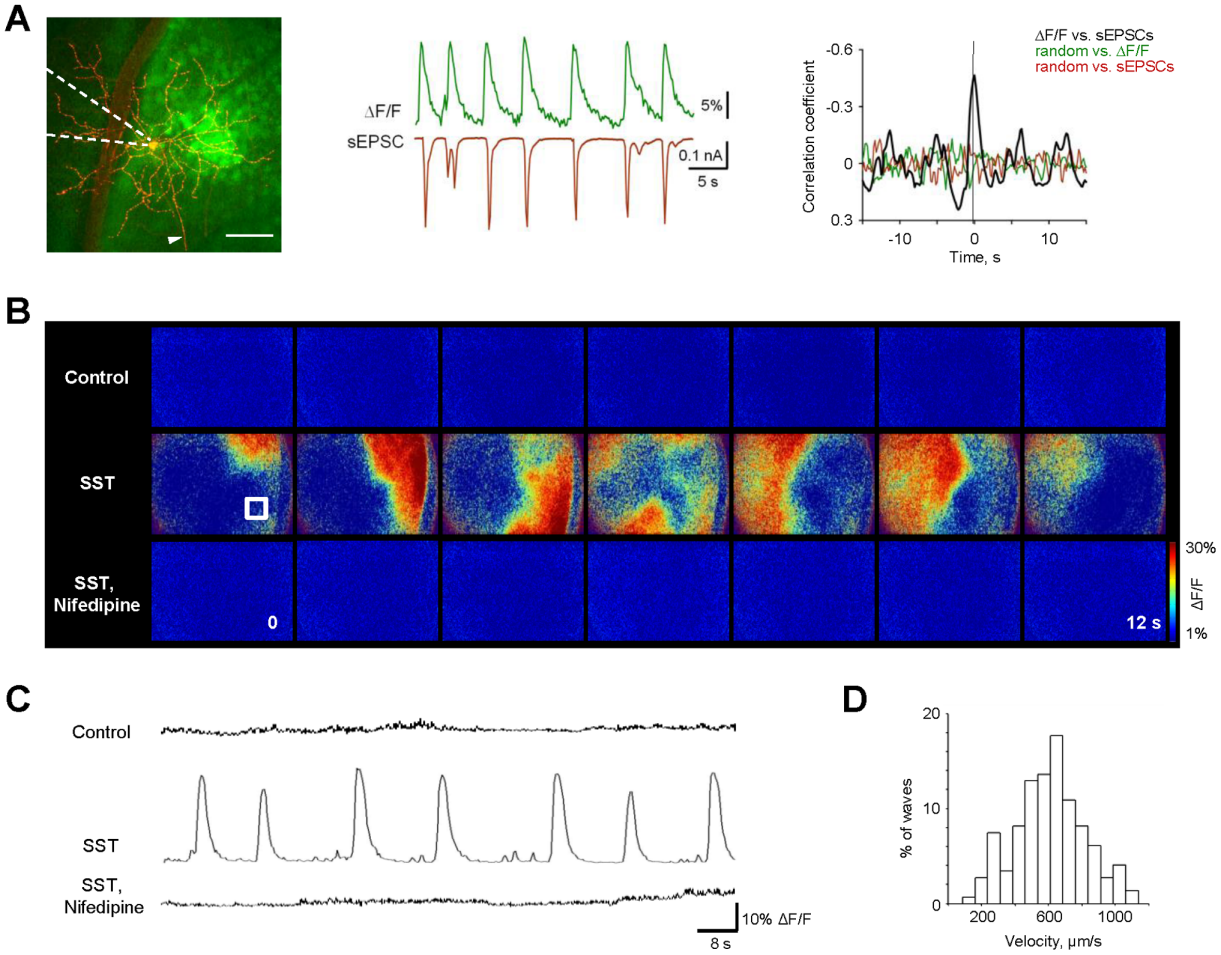
Calcium imaging reveals wave-like activity in adult retina that is suppressed by an inhibitory network. A: left panel, A fluorescence image showing loading of calcium indicator OGB-1-AM (green) with superimposed GC (red). Dashed lines indicate position of the recording pipette. Arrowhead points to GC axon. Dark shadow in the left is a blood vessel. Scale bar 80 µm. (A, middle panel) Simultaneous calcium imaging (top) and whole-cell sEPSC traces from GC (bottom). (A, right panel) Cross-correlations between ΔF/F and sEPSCs (right, n = 21) and randomized controls. Note inversed Y-axis to reflect the opposite directions for correlated events – each increase in intracellular calcium (positive) coincides with an inward current (negative). B: A sequence of ΔF/F pseudo color time-lapse images during 12 s of retinal activity viewed from GC side at P47. Blockade of inhibitory feedback reveals the wave-like propagating activity across the retina (middle). This activity is not evident in untreated control conditions (top) and eliminated by application of nifedipine (bottom), a selective antagonist of L-type VGCaCs. Size of the retinal region in each panel is 636× µm. C: Time course of the ΔF/F monitored over 120 s from retinal region marked by a white box in B at various conditions. D: Histogram of wave velocities under SST condition. Bin size is 100 µm/s, n = 130 of waves and n = 4 of retinas. See Supplementary Videos S1–4 for calcium recordings during control, SST and nifedipine conditions in raw (Videos S1, S2) and ΔF/F pseudo color modes (Videos S3, S4) from P47 retinas.

In the developing retina, the bursting of individual cells correlate across a large population to produce propagating activity known as retinal waves [9], [31], [32], [33]. To test whether the spontaneous events we revealed in adulthood correlate similarly, we used calcium imaging to observe network activation in adult retina. First, we established the correspondence between synaptic inputs (individual cell currents) and network activation (multicell calcium signals). We used voltage-clamp to record spontaneous excitatory postsynaptic currents (sEPSCs, Vhold = -60 mV) while simultaneously monitoring increases in calcium (ΔF/F) in the recorded GC and its surrounding region (Fig. 3A, left). Cells in the GC layer of P34–P90 mouse retinas were labeled with the calcium indicator Oregon Green BAPTA-1 AM (OGB-1 AM) using the multicell bolus loading method [16], [19], [24], [34]. Recorded GCs were identified as described in previous sections. Each increase in intracellular Ca^2+^ corresponded to a compound sEPSC (Fig. 3A, middle). Cross-correlation shows the time relationship of the two signals for a given cell, with a negative peak at time zero indicating a synchronized increase in calcium activity with sEPSC influx (Fig. 3A, right; peak r = −0.46, p<0.0001, n = 21 events. Across n = 10 cells, r = –0.42+/−0.09). Hence, sEPSCs observed in single cells are comparable with activity measured with OGB-1 AM.

To monitor population activity across the retina, calcium indicator was injected in 4–5 locations spaced by ∼400 um, and ΔF/F was observed at a lower magnification. Blockade of inhibition with SST revealed wave-like propagating activity across the retina (Fig. 3B, and Videos S2, and S3). This spontaneous correlated activity was not evident in control conditions (Fig. 3A and C, top, Video S1) and was eliminated by application of nifedipine (Fig. 3B and C, bottom, Video S4). The velocities of the propagating activity ranged from 139.36 to 1128.8 µm/sec (608.03±17.404 µm/sec, n = 147 waves, n = 12 retinas, P21–P85, Fig. 3D). The initiation rate of propagating waves was lower than that observed in both the regenerative potentials in individual BCs and oscillatory events in ganglion and amacrine cells (Table 1). This is not unusual, as each individual event may not necessarily translate into a propagating wave [24], [32]. In addition, individual events may also occur outside of a time window for larger coordinated activity [12], [35]. These experiments demonstrate that depriving the adult retina of inhibitory inputs reveals rhythmic events in individual cells, which in turn coordinate across a large population of GCs to generate propagating wave activity. Next, we investigated mechanisms governing wave propagation.

**Table 1:** Interevent intervals of developmental and pharmacologically-induced compound sEPSCs.

	control		DHβE		SST		nifedipine	
	x ± SD, s	incidence	x ± SD, s	incidence	x ± SD, s	incidence	x ± SD, s	incidence
**P5–6**	68.92±5.70	6(6)	na	0(6)	na	0(6)	na	0(6)
**P8–9**	107.78±55.25	6(6)	143.26±53.49	6(6)	107.41±58.15	6(6)	130.71±8.89	1(6)
**P12–14**	33.53±15.30	6(6)	30.86±18.91	6(6)	5.00±1.06	6(6)	27.93±79.65	2(6)
**P15–16**	48.19±30.62	2(6)	27.78±22.27	1(6)	2.67±1.29	6(6)	na	0(6)

### Intercellular Mechanisms Contributing to Adult Retinal Waves

Multiple mechanisms drive retinal waves at different developmental stages [2]. Similar to our findings in adulthood, stage III waves are shown to rely on glutamatergic signaling, though the mechanisms involved in their propagation are not clear. Gap junctions continue to play a role in late-stage retinal waves [7], [8]. In the adult retina, gap junctions mediate lateral GC-AC and GC-GC interactions [36], and also connect cone BCs to AII ACs [37]. Voltage-gated sodium channels mediate the output of both BCs and AII ACs [38], [39]. Additionally, glutamate spillover significantly effects the initiation, propagation, and synchronization of stage III waves [24], [40]. We wanted to determine the contribution of these mechanisms to induced wave-like activity in adult GCs, using pharmacological blockers of voltage-gated sodium channels, excitatory amino acid transporters (EAATs), and gap junctions.

**Figure 4:**
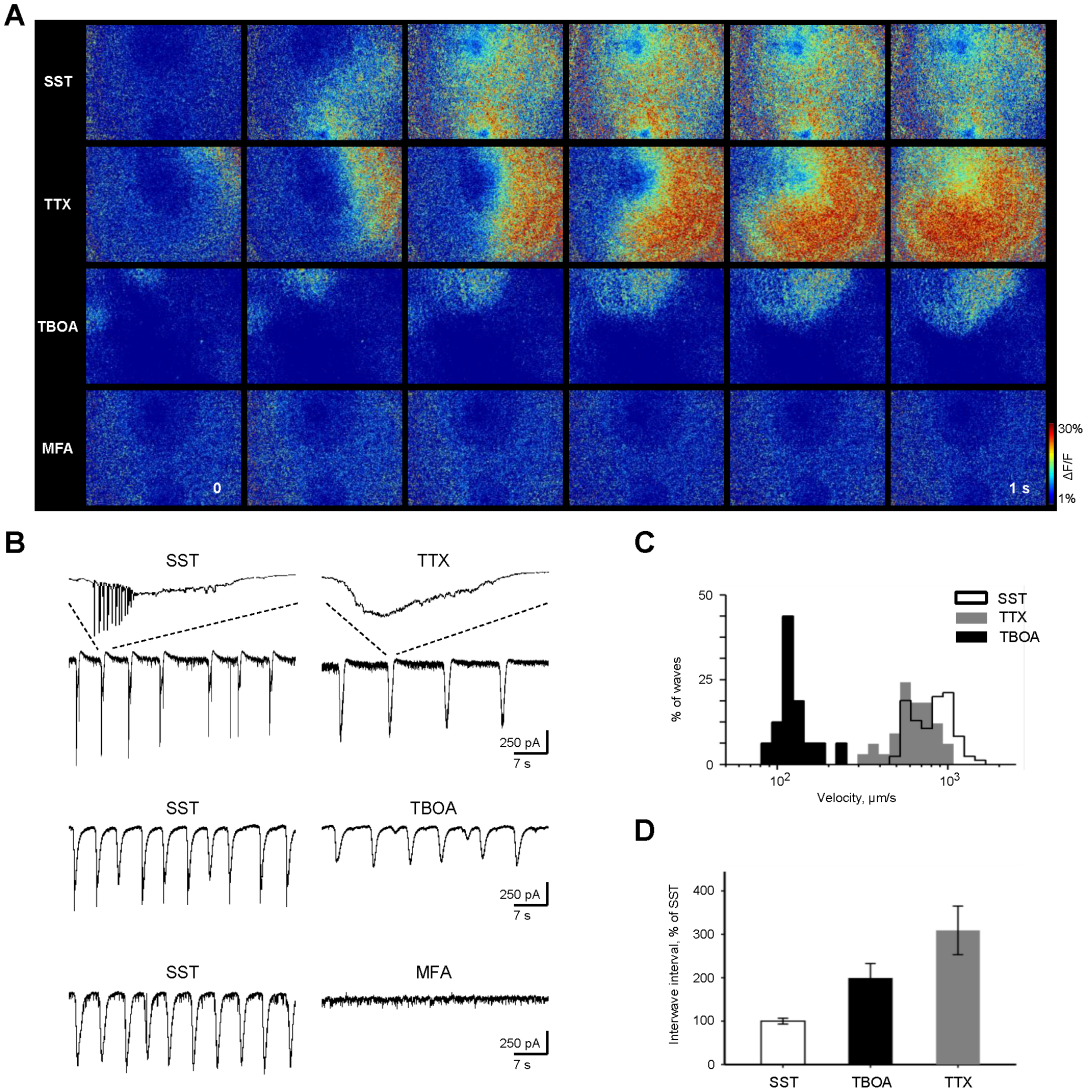
Contribution of sodium channels, glutamate spillover and gap junctions to propagation of adult retinal waves. A: ΔF/F pseudo colored time-lapse images showing 1 s of retinal activity under different pharmacological conditions, illustrating the contribution of voltage-gated sodium channels, glutamate transporters, and gap junctions, respectively, to SST-induced adult retinal waves. Size of the retinal region in each panel is 636×1130 µm. B: Compound sEPSCs recorded from GCs under different pharmacological conditions. In the top panel, the inset highlights the selective blockade of spiking activity following application of TTX. For illustrative purposes, these recordings were obtained using a recording pipette with higher input resistance, to reveal spiking activity along with currents. Compound sEPSCs persisted under TTX and TBOA, but were abolished under MFA. C: Cumulative histogram of wave velocities under SST (transparent bars, black outline), TBOA (black bars), and TTX (gray bars). Velocities following TBOA and TTX are significantly slower than under SST alone (ANOVA, Bonferroni post-hoc tests, p>0.0001 and p = 0.0067, respectively). D: Bar graph of interwave intervals under different pharmacological conditions. Intervals following TBOA and TTX are significantly longer than under SST alone (ANOVA, Bonferroni post-hoc tests, p = 0.0016 and p>0.0001, respectively). All data are reported as means ± SEM. See Supplementary Videos S5-88 for ΔF/F pseudo color recordings of the effect of TTX, TBOA, and MFA on waves.

The effects of these individual agents on adult retinal waves are summarized in Fig. 4. We first blocked voltage-gated sodium channels using TTX (2 µM). Wave velocity was slightly reduced (637.50±28.95 under TTX+SST vs 820.00±25.77 with SST alone, p = 0.0067, n = 34, ANOVA with Bonferroni post hoc tests) and the waves were far less frequent (3.09±0.56 times SST, p<0.0001), but still present. Next, to determine the role of glutamate spillover, we applied TBOA (25 µM), a nontransportable blocker of EAATs. Under these conditions, wave velocity is slowed by nearly a log unit (154.84±7.12 under TBOA+SST vs 820.00±25.77 with SST alone, p<0.0001, n = 38), which is similar to the 150 µm/s observed by Blankenship et al. (2009) following the application of the same concentration of TBOA to stage III retinal waves. Interwave intervals were increased slightly (1.99±0.34 times SST, p = 0.0103). Finally, when gap junctions were blocked with meclofenamic acid (MFA, 200 µM), a potent gap junction antagonist [41], waves were abolished. Together, these findings suggest that interactions between cone BCs and AII ACs play a large role in propagation, but do not mediate it entirely. Instead, propagation may occur through GC-GC and GC-AC gap junction connections, and may be tuned by glutamate spillover.

### Developmental Changes in Spontaneous Rhythmic Activity

The effects of neurotransmitters change as the retina matures. GABA and glycine initially mediate excitatory signaling, but transition to having inhibitory actions [14], [15]. In parallel, retinal waves transition from being primarily mediated by acetylcholine (stage II waves) to glutamate (stage III waves), and remain glutamatergic until waves disappear [2]. We hypothesize that the maturation of the inhibitory network gradually obscures glutamatergic waves. Thus, we tracked the interaction between wave-generating excitatory networks and wave-suppressing inhibitory networks as the retina matures.

**Figure 5:**
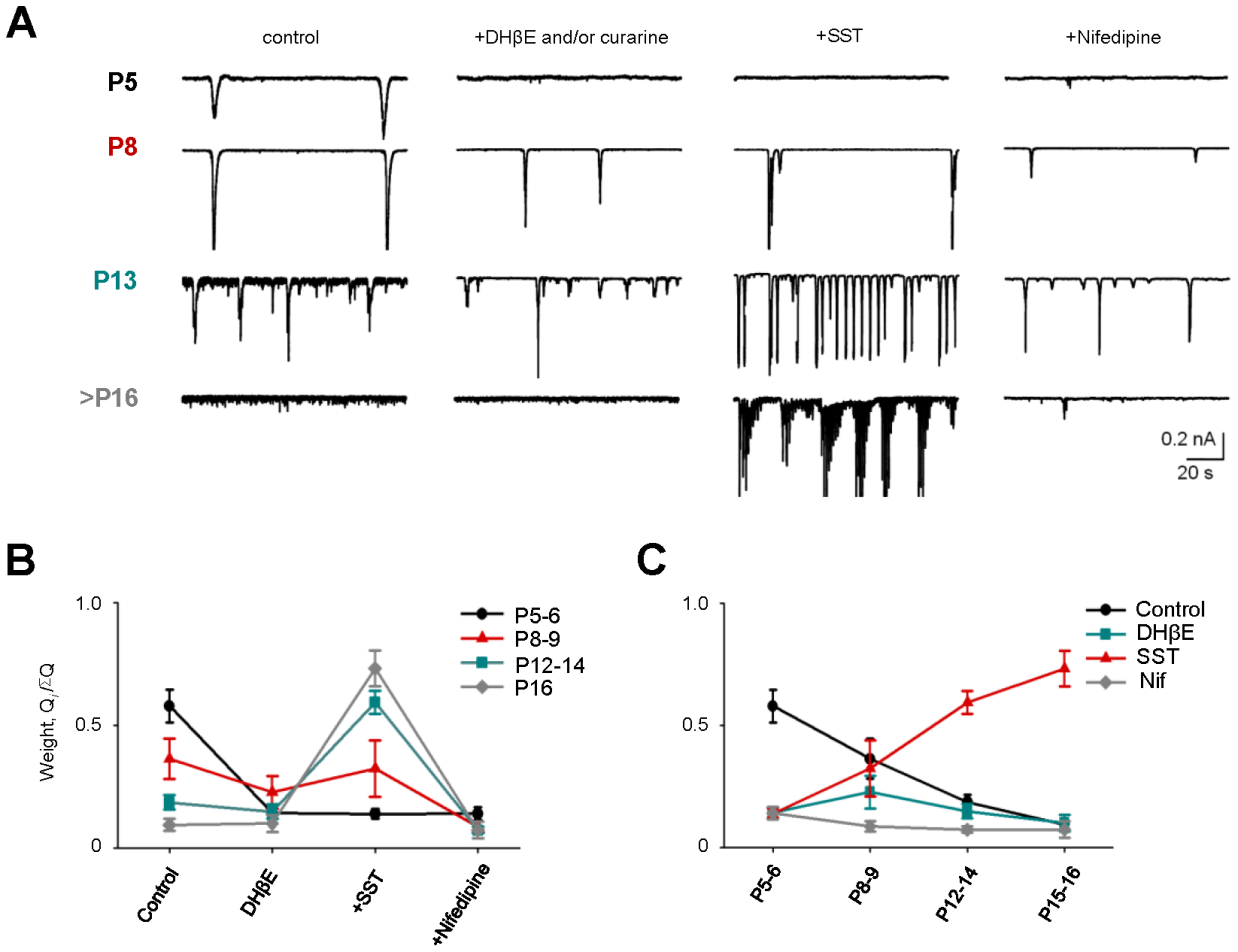
Developmental changes in the effect of cholinergic, inhibitory and L-type voltage-gated calcium channel-dependent networks on spontaneous activity. A: sEPSCs recorded from GCs at different ages in control and following the blockade of nicotinic AChRs, inhibitory transmission and L-type VGCaCs. B and C: Summary graphs of GC activity and the contribution of different receptors over age. Early in postnatal development (P5–6) the activity is mediated by AChRs and blocked by DHβE/curarine. At second postnatal week, the contribution of AChRs is reduced. In contrast, regenerative compound activity revealed by SST is more prominent with age, as BC axons and inhibitory inputs mature and spontaneous currents decrease (C, red and black traces). This transition is most prominent in late second postnatal week (see text and Table 1 for details). All data are reported as means ± SEM.

To separate the contribution of these distinct networks to progressive stages of retinal waves, we monitored sEPSCs under the following pharmacological conditions – DHβE/curarine (100 µM/50 µM), SST, and nifedipine – to block cholinergic and inhibitory transmission, and BC-driven glutamatergic input to GCs, respectively (Fig. 5A). We found that antagonists had significantly different effects on the total charge transfer of sEPSCs at different ages (two-way repeated measures ANOVA, main effect of drug, p<0.0001, significant interaction with age, p<0.0001, n = 6 cells for each age group; Fig. 5B). Early in postnatal development (P5–6), wave-associated compound sEPSCs were blocked by DHβE/curarine and were not sensitive to SST or nifedipine, consistent with a primary role for cholinergic transmission, along with the non-inhibitory effects of GABA/glycine at this stage [14], [23]. Further in development (P8–P14), waves become less sensitive to DHβE/curarine and more sensitive to SST and nifedipine, consistent with a decreasing role for cholinergic transmission and a growing contribution of both glutamatergic transmission and the inhibitory network. SST reveals progressively larger wave-associated compound sEPSCs, suggesting that (1) intrinsic BC output increases as glutamatergic inputs mature, and (2) at the same time, suppression of BC output increases as inhibitory inputs mature. After eye opening (>P16), wave-associated compound sEPSCs are not present in control conditions, consistent with the paucity of retinal waves at this stage [33]. Similar to adult retina, these sEPSCs re-emerge following the block of inhibitory transmission with SST, and are silenced again by nifedipine. Overall, there is an inverse relationship between the progressive disappearance of wave-associated compound sEPSCs in control conditions (ANOVA, p<0.0001; post hoc linearity test, m = –0.0814, r^2^ = 0.6621, p<0.0001, n = 6 cells for each age group; Fig. 5C, black trace) and the increase in sEPSC charge transfer in the presence of SST (ANOVA, p<0.0001; post hoc linearity test, m = 0.1024, r^2^ = 0.6596, p<0.0001, n = 6 cells for each age group; Fig. 5C, red trace). Together, these data suggest that maturing glutamatergic connections between bipolar and postsynaptic cells enhance intrinsic oscillatory activity, which is suppressed by the inhibitory network that matures in parallel. It also suggests that the mechanisms responsible for late-stage developmental waves may exist in adulthood.

### Distinct Inhibitory Components Uniquely Shape Spontaneous Rhythmic Activity

We have demonstrated that blocking the inhibitory network reveals intrinsic rhythmic activity, driven by BCs (Fig. 1), that propagates across the adult retina (Fig. 3, Videos S2, C). However, the inhibitory network mediating BC-to-GC transmission contains three major receptor types with distinct kinetics: GlyRs and GABA_A_Rs decay quickly, while GABA_C_Rs have a significantly slower decay time [25], [42], [43], essentially acting as low- and high-pass filters, respectively. Therefore, to determine whether these receptors play distinct roles in shaping the temporal properties of spontaneous regenerative BC activity, we monitored postsynaptic currents while sequentially blocking individual receptor types.

**Figure 6:**
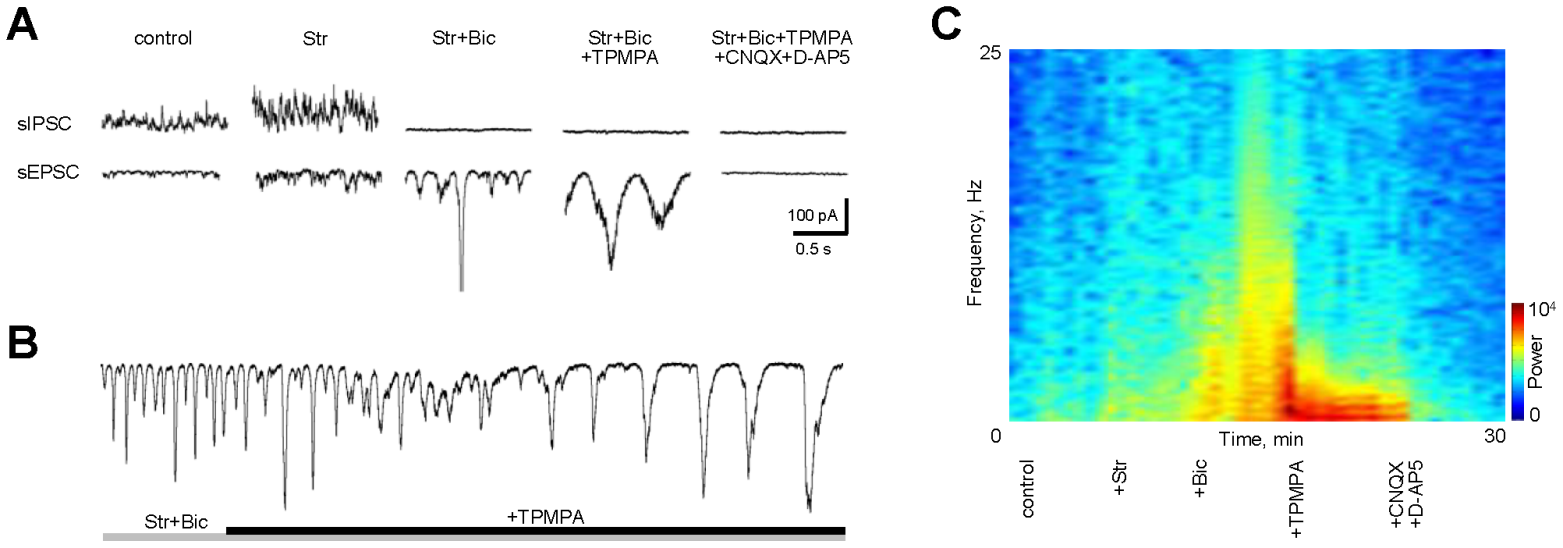
Inhibitory network tunes the frequency of oscillatory activity. A: sIPSCs and sEPSCs from GCs at different conditions. Gradual removal of distinct components of inhibitory network tunes the frequency of BC-driven oscillatory activity in GC. Blockade of glycine (strychnine) and GABA_A_Rs (SR-95531 or bicuculline) shaped the oscillatory activity into high-frequency and low-amplitude events. The removal of slow inhibitory inputs mediated by GABA_C_Rs (TPMPA) shaped the oscillator into low-frequency and high-amplitude mode. B: A representative whole-cell sEPSC recording from a GC showing a gradual transition during a blockade of ‘fast’ and ‘slow’ components of inhibitory inputs. C: Response spectrogram illustrating transition in power and frequency profiles of BC output FFT power for GC shown in B. Blue to red transition depicts increase in FFT power at specified frequency domain during different pharmacological conditions.

First, GlyRs were blocked with strychnine, increasing sEPSC frequency (p<0.01, n = 6, repeated measures ANOVA, Tukey post-hoc test), without a significant change in FFT power (p = 0.11, n = 6, Fig. 6A). This suggests that regenerative activity is not primarily mediated by glycinergic transmission. Next, GABA_A_Rs were blocked with bicuculline (100 µm), resulting in large and frequent regenerative events (p = 0.04, n = 6, Fig. 6A). Finally, GABA_C_Rs were blocked with TPMPA, reducing event frequency while increasing power (p<0.01, n = 6). The transition is gradual (Fig. 6B), and can be observed as a change dominant frequency in a power spectral analysis (Fig. 6C). All sEPSCs were blocked following the addition of CNQX and D-AP5, confirming that they originated from BCs and were transmitted synaptically.

**Figure 7:**
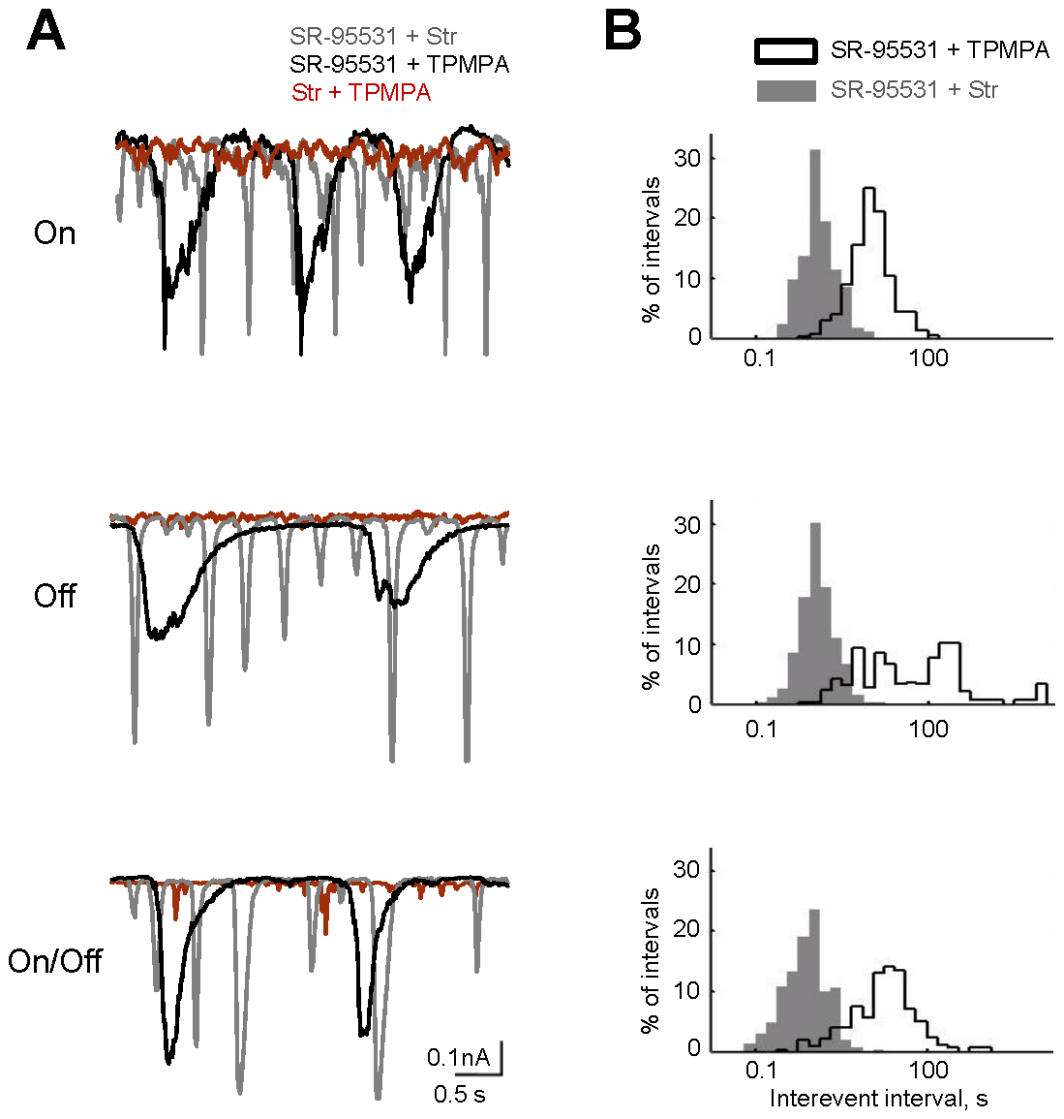
Individual inhibitory receptors differently regulate spontaneous events. A: Representative sEPSCs from major classes of GCs at individual conditions. B: Distributions of interevent intervals for the traces shown in A. In this experiment, the dual blockade of inhibitory receptors paradigm is applied to reveal the contribution of an individual receptor types. GABA_C_Rs filter out slow events, allowing mostly high-frequency events (<10^3^ ms) to pass. GlyRs filter out fast events, allowing mostly low-frequency events (>10^3^ ms) to pass. Events do not occur when GABA_A_Rs are active.

Our findings suggest that different receptors of the inhibitory network distinctively mediate frequency components of spontaneous regenerative activity. To further determine this, individual components were pharmacologically isolated, and the remaining activity was examined across conditions. Pairs of antagonists were applied to leave a single receptor type active (Fig. 7). When only GABA_C_Rs were active (SR-95531 + strychnine), events occurred more frequently than with SST (Kolmogorov-Smirnov test, p<0.0001, n = 16 cells), confirming that GABA_C_Rs act as a high-pass filter. When only GlyRs were active (SR-95531 + TPMPA), events occurred less frequently than with SST (Kolmogorov-Smirnov test, p<0.0001, n = 13 cells), suggesting that GlyRs act as a low-pass filter. When only GABA_A_Rs were active (strychnine + TPMPA), no events occurred, suggesting that GABA_A_Rs act as a gating mechanism. These roles were similar across major cell classes. Together, this demonstrates that the individual components of the inhibitory network coordinate to regulate spontaneous regenerative activity from BCs.

## Discussion

We have revealed and examined wave-like activity in adult retina. These spontaneous glutamatergic events grow in magnitude as the retina matures, but are increasingly suppressed by the inhibitory network as it develops. This activity is differentially mediated by distinct receptors of inhibitory neurotransmitters. We provide evidence that the cellular properties that generate glutamatergic retinal waves may be preserved in adult retina, and that they are continuously suppressed by a mature inhibitory network.

### Wave-like Activity Persists in Mature Retina

Retinal waves play an important role in establishing visual circuits within the retina and beyond [44], [45], [46], [47], [48], [49], [50]. Waves progress through three stages, which are respectively mediated via three modes of transmission – (I) gap junctions, (II) nicotinic acetylcholinergic receptors, and (III) ionotropic glutamate receptors. Though waves disappear around the time of eye opening, we demonstrate that waves are generated by a persistent mechanism that extends into adulthood, suppressed by a mature inhibitory network.

We find that pharmacologically blocking the inhibitory network reveals propagating wave-like activity in adult retina (Fig. 3, Video S3). Several observations suggest that this activity resembles developmental retinal waves. Like stage III retinal waves, this activity is glutamatergic [22], [23], relying on excitatory input from BCs (Fig. 1) which induce rhythmic spiking activity in postsynaptic cells (Fig. 2). This activity also shows a dependence on L-type voltage-gated Ca^2+^ channels (Figs. 1–3), which play a role in wave propagation and potentiation during development [51], [52].

The propagating activity we observe suggests that the wave-generating mechanisms are resilient throughout development. This has been previously suggested, as mutants lacking stage II waves still exhibit gap junction-dependent events, indicating a persistence of mechanisms that mediate stage I waves [53]. Suppressing stage II waves with nAchR antagonists similarly reveals gap junction-dependent events [8]. Our data suggests that the propagation of adult retinal waves is reliant on gap junctions (Fig. 4). Previous studies have shown that glutamatergic waves in connexin knock-out mice do not require gap junctions to propagate [54]. Previous studies using gap junction blockers have had mixed results, ranging from increased wave frequency to blocked waves [12], [22], [51], however, these did not examine stage III waves. Recently, it has been shown that blocking gap junctions silences stage III waves [40]. The discrepancy between the presence of glutamatergic waves in connexin knockout mice and the silencing effect of gap junction blockers may be due to one or both of the following explanations [40]: (1) homeostatic adjustments preserve wave dynamics, which may include compensatory changes to iGluR expression or an alternative means of wave propagation, or (2) non-specificity of gap junction blockers may affect propagative mechanisms besides gap junctions.

Blocking EAATs with TBOA had seemingly unexpected effects on adult waves. Previous studies have shown that TBOA enhances EPSCs via spillover activation [55], but under SST conditions, TBOA reduces EPSC amplitude and slows wave velocity (Fig. 4). This can be explained by a cumulative effect of blocking both inhibition and glutamate reuptake. While individually, each of these conditions increases spillover, they have different mechanisms, which in tandem may not produce simply an additive effect. SST increases the rate of release from bipolar cell terminals, depleting neurotransmitter pool. EAAT-mediated reuptake replenishes this pool. When reuptake is blocked by TBOA, however, the amount of glutamate in bipolar cell terminal declines. This, in turn, leads to observed reduction in EPSC amplitude relative to SST alone. Additionally, it has been shown that TBOA slows down fast waves and speeds up slow waves [1], [24]. Blankenship et al. (2009) found that the velocity of Stage III waves following treatment with TBOA is approximately 150 um/s, which is the velocity we observed in adult retina following SST+TBOA treatment. Thus, spillover does not necessitate enhancement of wave-like activity, but rather seems to reduce interwave variability and promote synchronization [40].

Does spontaneous correlated activity serve a purpose in adult retina? Though waves are absent, selective transient activation of the underlying propagative mechanisms may still occur. This is evidenced in part by the observation of correlated firing in GCs in the adult retina of a number of species [33], [56], [57], [58]. Oscillations can also occur during responses to light, suggesting a role in mediating complex light responses. For example, during light-evoked responses – without pharmacological or genetic intervention – BCs and postsynaptic starburst amacrine cells exhibit oscillatory currents, indicating that this manner of activity may play a role to encode moving stimuli [5], [28]. Future studies are needed to better discern the role of this activity in adult retina.

### Balancing Excitation and Inhibition in the Retina

Several studies have associated the disappearance of waves to the development of the retinal inhibitory network [7], [8]. GABA and glycine receptors are initially excitatory, but become inhibitory as the retina reaches maturity [14], [15] due to a change in the chloride equilibrium potential which coincides with an up-regulation of the chloride extruder KCC2 [59]. This is a gradual process, occuing throughout the course of stage II and stage III waves [14], [60]. In ferret, it has been shown that GABAARs become inhibitory after P18, while glycine receptors become inhibitory by P21 [10], ages where stage III waves are still active. During stage III waves, GABA_A_ and GABA_C_R activation suppresses retinal wave frequency, while GABA_A_ and GABA_C_R blockade leads to an increase in wave frequency [8], [15]. Several studies have also suggested that GABA_A_Rs, in particular, to an evolving mechanism for homeostatic control over spontaneous retinal activity; GABA_A_R agonists blocked postnatal waves entirely, while GABA_A_R antagonists progressively increased wave size with age [11], [61]. This provided initial evidence that components of the wave-generating circuitry are kept in check by establishing a balance between excitation and inhibition.

**Figure 8:**
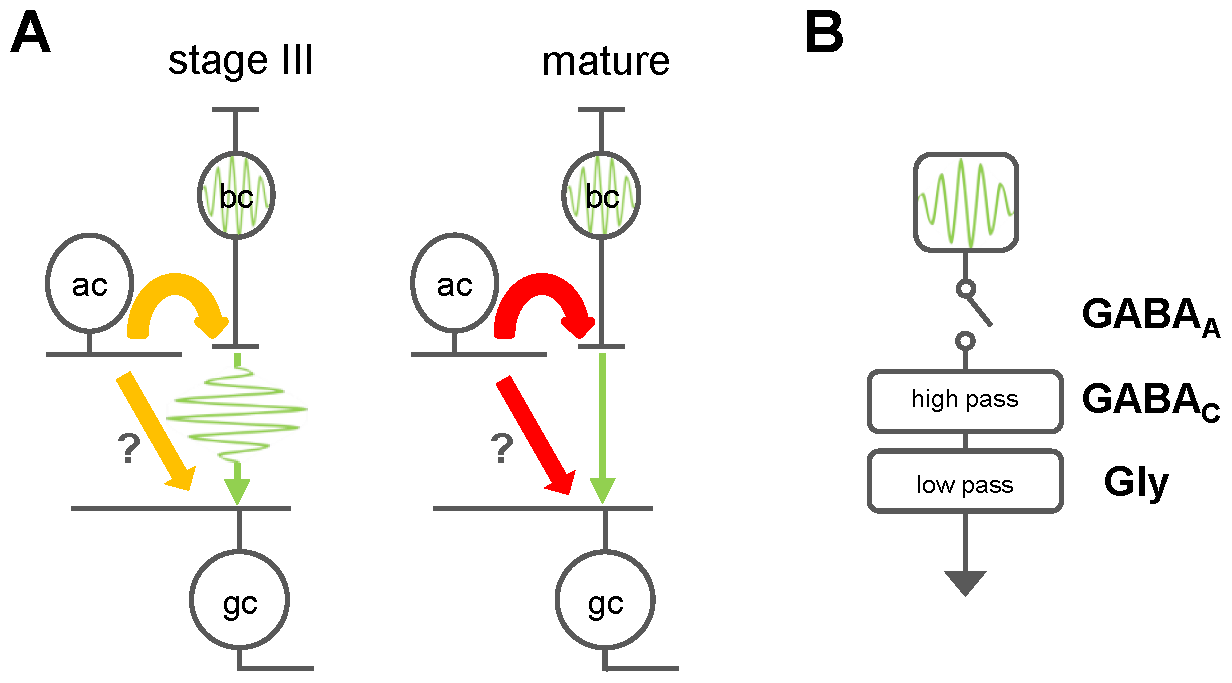
Spontaneous regenerative bipolar cell activity persists through adulthood and is suppressed by inhibitory inputs. A: Stage III developmental waves are similar to the pharmacologically-unveiled wave-like events in mature retina. During stage III waves, inhibitory inputs are not fully matured (orange arrows), and permit the generation of glutamatergic events. Inhibitory synaptic inputs silence these spontaneous glutamatergic events in mature retina (red arrows). Precise contributions of presynaptic and direct components of inhibitory network remain unclear. B: Schematic of inhibitory receptor contributions in modifying regenerative events. GABA_A_Rs are the main suppressor of oscillatory events and are proposed to act as gating mechanism. GABA_C_Rs act as a high-pass filter. GlyRs act as a low-pass filter. The diagram does not necessarily depict the sequence of processing events.

We propose that BCs intrinsically oscillate, conferring glutamatergic rhythmic activity to postsynaptic cells. During stage III retinal waves, this activity is relatively unchecked, partly due to the immaturity of the inhibitory network (Fig. 8A, left). GABA/glycine receptor-mediated activity does not simply switch from excitatory to inhibitory; rather, it is a gradual process that occurs throughout the course of stage II and stage III waves [14], [60]. In ferret, it has been shown that GABAARs become inhibitory after P18, while glycine receptors become inhibitory by P21 [10], ages where stage III waves are still active. Also crucial is that bipolar cell terminals are still developing during this age – for example, mouse bipolar cell ribbon synapses do not appear before p11 [62], shortly after stage III waves begin [2]. Similarly, in ferret, the area and number of bipolar cell terminals increase throughout the period of stage III waves [63]. Once the retina is mature, BCs are inhibited so that these spontaneous oscillations are silenced (Fig. 8A, right); however, we demonstrate that blocking inhibition reveals these oscillations (Fig. 1–3). Blocking glutamatergic photoreceptor input with L-AP4 increases interevent intervals and the size of oscillations, but the initiation is ultimately dependent on blocking inhibitory transmission, which is consistent with previous observations that visual experience is not necessary for the disappearance of waves [33]. Additionally, this suggests that mGluR6 receptors, which inhibit ON bipolar cells via hyperpolarization, may also play a role in wave suppression. Indeed, in the nob mouse mutant, which has a defect in the mGluR6 signaling cascade within bipolar cells, waves persist into adulthood, perturbing the organization of retinogeniculate projections [35]. This supports the idea that the balance of excitation and inhibition onto BCs mediates the initiation rate of glutamatergic retinal waves [24], and shows that this balance is shifted as the retina matures to preclude the formation of spontaneous wave-like activity. However, these recordings were performed using whole-cell patch clamp, which can affect the second messenger system and thus mGluR6 function; therefore, perforated patch recordings would provide a clearer picture of the role of mGluR6 in this activity.

If the inhibitory network regulates spontaneous correlated BC output, how does this compare to its role in shaping light responses? Inhibitory network interactions contribute to a variety of features of visual responses, including temporal and spatial representations, signal gain, dynamic range, and contrast sensitivity [64], [65], [66], [67], [68], [69], [70], [71]. Temporal properties of light responses are conferred, in part, by the respective kinetics of individual classes of inhibitory receptors [reviewed by 25]. Here, we demonstrate that inhibitory receptors also modulate spontaneous regenerative BC activity, and that individual receptor types have different roles (Fig. 7). GABA_A_ receptors act as a gating mechanism, preventing spontaneous compound release from BCs, while GABA_C_ and glycine receptors act as high pass and low pass filters, respectively (Fig. 8B). We propose that distinct localization patterns of inhibitory receptor components across the retinal network may also play a critical role in shaping inputs to GCs. It remains to be evaluated the precise roles for presynaptic, direct and serial inhibition in modulating the regenerative activity of BCs. Nevertheless, we find that inhibition plays a complex role not only in mediating light-evoked signal transmission, but also in regulating spontaneous bipolar-to-ganglion cell transmission.

In conclusion, while the re-emergence of wave like activity in adulthood can have detrimental effects on visual processing, the persistence of wave-generating machinery is potentially advantageous. Retinal waves play an instructive role in development, aiding in the formation of visual circuits. The ability to recapitulate this process could be incorporated into repair strategies for blinding disorders that cause cell loss and network remodeling in the retina. Future studies are under way to shed light on this phenomenon and its interactions within both healthy and diseased tissue.

## Supporting Information

Video S1 **Calcium imaging reveals wave-like activity in adult retina.** A: Unmodified real-time movie of fluorescence imaging of GC layer in P47 retinal wholemount loaded with OGB-1 AM in control conditions. Individual cells are distinguishable and no correlated activity is evident. 100× magnification.
(MP4)

Video S2 **Block of inhibitory transmission of the same preparation with SST unveils correlated activity that propagates in waves across the retina and is clearly visible in raw video at 100× magnification.**
(MP4)

Video S3 **The same preparation at 20× magnification with changes in intracellular calcium (ΔF/F) mapped to a color gradient and overlaid on the original video frames.**
(MP4)

Video S4 **Adult retinal waves are blocked by nifedipine. 20× magnification.**
(MP4)

Video S5 **Contribution of sodium channels, glutamate spillover and gap junctions to propagation of adult retinal waves. Videos S5–S8 are pseudo colored ΔF/F at 20× magnification.** See also Fig. 5. Block of voltage-gated sodium channels with TTX greatly increases interwave intervals and slightly reduces velocity.
(MP4)

Video S6 **Block of excitatory animo acid transporters with TBOA increases interwave intervals and greatly reduces velocity.**
(MP4)

Video S7 **Block of gap junctions with MFA abolishes waves.**
(MP4)

Video S8 **Applying MFA before SST prevents waves from appearing.**
(MP4)
